# Stylized facts of intraday precious metals

**DOI:** 10.1371/journal.pone.0174232

**Published:** 2017-04-27

**Authors:** Jonathan Batten, Brian Lucey, Frank McGroarty, Maurice Peat, Andrew Urquhart

**Affiliations:** 1 Monash University Business School, Monash University, Caulfield, Victoria, Australia; 2 Trinity Business School, Trinity College Dublin, Dublin, Ireland; 3 Southampton Business School, University of Southampton, Southampton, United Kingdom; 4 University of Sydney Business School, Sydney, New South Wales, Australia; 5 Southampton Business School, University of Southampton, Southampton, United Kingdom; East China University of Science and Technology, CHINA

## Abstract

This paper examines the stylized facts, correlation and interaction between volatility and returns at the 5-minute frequency for gold, silver, platinum and palladium from May 2000 to April 2015. We study the full sample period, as well as three subsamples to determine how high-frequency data of precious metals have developed over time. We find that over the full sample, the number of trades has increased substantially over time for each precious metal, while the bid-ask spread has narrowed over time, indicating an increase in liquidity and price efficiency. We also find strong evidence of periodicity in returns, volatility, volume and bid-ask spread. Returns and volume both experience strong intraday periodicity linked to the opening and closing of major markets around the world while the bid-ask spread is at its lowest when European markets are open. We also show a bilateral Granger causality between returns and volatility of each precious metal, which holds for the vast majority subsamples.

## Introduction

This paper examines the intraday periodicity, correlation and volatility interaction in four precious metals markets. Our data set covers over 15-years of 5-minute data on gold, silver, platinum and palladium and finds significant evidence of intraday periodicity in returns, volatility, trading volumes and bid-ask spreads as well as strong evidence of bilateral Granger causality between returns and volatility. As well as being important in its own right in explaining high-frequency precious metal dynamics and trading behaviour, intraday periodicity, correlation and volatility interaction have important implications for investors trading precious metals intraday.

Gold is one of the most intensively traded assets, a feature not often understood by market participants. In 2011, estimated daily international turnover in gold was of the order of 4,000 metric tons, equivalent to a then average value of over $240 billion. This is approximately the same as the daily dollar volume of trade on all of the world’s stock exchanges combined [1]. If, as is common, we consider gold as a currency its turnover exceeds that in all but four currency pairs [2]. Gold trading is also highly concentrated, as it is in the foreign exchange market, with the two major centers for gold trading, London (physical, over-the-counter (LOTC) spot trade) and the New York Mercantile Exchange Futures Market (COMEX), totaling 85% (78.0% and 7.7% respectively) of global turnover value [3]. We study gold, silver, platinum and palladium since they are the most traded due to them having ISO-4217 currency codes which means they are traded as a currency, see http://www.iso.org/iso/home/standards/currency_codes.htm

Our study is motivated by the fact that most financial time series exhibit periodicity. With high-frequency data the problem of periodicity becomes more complex since the entire form of the daily activity has to be taken into account. There is widespread empirical evidence that trading patterns vary systematically over the trading day and these patterns are highly correlated with intraday variations in returns, volatility, volume and bid-ask spreads in stock markets (for example see [4], [5], [6], [7], [8]), Foreign Exchange (FX) markets (see for example [9], [10], [11]) and Exchange Traded Funds (ETFs) (see for example [12]).

The finance literature has also shown that most intraday trading activity exhibits a U-shaped pattern, (for example [13] for Toronto stock exchange; [14] for the New York Stock Exchange (NYSE); [15] for the Tokyo stock exchange), while UK markets experience a M-shape where the volume is higher around the opening of US markets, see [16] and [17]. Also, [5] document a reverse J-shaped pattern of NYSE quotations and [18] support this pattern with London Stock Exchange (LSE) intraday spreads. Elevated opening and closing returns have been reflected in the volatility patterns, where a U-shape is found by [13], [19], [20] and [21].

This paper fills three lacunae in the literature. First, despite the extensive literature on periodicity in stock markets and FX markets, there is a notable lack of studies examining the periodicity of precious metals. [22] study the main stylized facts and dynamic properties of spot precious metals from 27^th^ December 2008 to 30^th^ November 2010 at 5-minute and 50-minute frequencies. They find clear evidence of periodic patterns matching the trading hours of the most active markets round-the-clock and therefore conclude that precious metals spot returns have similar properties to those of traditional financial assets. [23] examine the 5-minute gold futures market and find periodicity in absolute returns and the returns movements in response to macroeconomic announcements. [24] study the dynamic behaviour of six commodities, including gold, and find that intraday returns have long memory. [25] study high-frequency futures data for gold, silver and copper from 1999 to 2008 through four measures of volatility and find that each of the return distributions are not normal. [3] examine the gold markets and find intraday periodicity in the context of how the London and New York markets interrelate. Given the size of the market, there remains a lack of studies examining the intraday periodicity of precious metals spot rates, this study seeks to fill this gap.

Second, a further gap in the literature revolves around the well-known stylised fact in finance that stock index returns are negatively correlated with changes in volatility [26]. This distinctive cross dependence pattern plays an essential role in the development of volatility as an asset class, in modelling volatility and in option pricing. Many studies have examined this phenomenon in stock markets, see [27], [28], [29], [30], [31]. However, there are to our knowledge no extant studies that study this relationship in precious metals at a high-frequency.

A third gap relates to the evaluation of intraday features in over the counter trades. By contrast to futures markets, where there is a great deal of research across a large number of assets, over the counter markets have received much less attention. In the area of gold the only comparable study to this paper is that of [32]. A frequent assumption of over the counter market analysis is that the over the counter market is illiquid see [33] and [34]. That is not the case here.

Transparency in the OTC markets are typically rather low. There is no public record of trade volumes or prices, only the quotes are observable. For gold, this lack of transparency was the genesis of the Loco London Liquidity Survey [35] which has gone some way to demonstrate gold as a liquid asset. Evidence in this paper on bid-ask spread and volume will therefore be of use to fill this gap.

This paper considers the intraday patterns in the returns, volatility, volume and the bid-ask spread of gold, silver, platinum and palladium at a 5-minute frequency from May 2000 to April 2015. The intraday patterns of precious metals have not received detailed empirical attention in the literature, which is all the more surprising given the growth of precious metals as investment assets as well as the growth of high-frequency trading. This paper also investigates the lead-lag relationship and Granger causality between returns and volatility of precious metals at high frequency, an area currently unexplored.

Therefore, this study contributes to the literature in a number of ways. Firstly, this is the first study to examine the stylized facts of all precious metals at high frequency over a long sample period. [22] study high-frequency precious metals from December 2008 to November 2010, which may not be the best time to determine the stylized facts of precious metals given the aftermath of the financial crisis. Secondly, by splitting our data into three equally-sized subsamples, we also study how the stylized facts of precious metals have developed over time in a dynamic framework. Thirdly, we document the intraday periodicity of precious metals which can offer valuable information to investors trading precious metals. Fourthly, we also study the relationship between returns and volatility of high-frequency precious metals, which has been unexplored in the empirical literature.

The remainder of the paper is organized in as follows. The next section presents the data and methodology while Section 3 reports the empirical results. Section 4 reports the empirical results while Section 5 summarises the findings and provides conclusions.

## Material and methods

The data is collected from Thomson Reuters Tick History for the period 1^st^ May 2000 to 30^th^ April 2015 and consist of the closing prices, time stamp, the bid/ask price, and the number of trades for gold, silver, platinum and palladium. These prices are made by wholesale market practitioners with prices and trades time-stamped as they arise in online trading platforms.

In order to examine the periodicity of these precious metals, it is important to use short enough intervals to capture the high frequency behaviour of the data, but at the same time long enough to avoid any undue noise [36]. Therefore, we follow [37] who suggests that 5-minute intervals are the best compromise. The markets of all four precious metals trade from Sunday 22.00 to Friday with a daily break between 21.00 and 22.00 GMT. We filter the data by removing any errors caused by missing bid/ask data and also remove any data when the market is closed.

Given our large sample period, the increased attention to precious as an investment and attention in the academic literature, the stylized facts may change over our 15-year full sample period. Therefore as well as studying the full sample period, we also split our sample into three equal-sized subsamples, from 1^st^ May 2000 to 30^th^ April 2005, 1^st^ May 2005 to 30^th^ April 2010 and 1^st^ May 2010 to 30^th^ April 2015.

The variables of interest in this paper are returns, volume, volatility and the bid-ask spread (BAS). From 5-minute transaction prices of each precious metal, we calculate the return following [38] such that;

$$r_{t,d}=\left(ln\;\;CP_{t,d}-ln\;CP_{t,d-1}\right)\times 100$$ (1)

where *r*_*t*,*d*_ is the return for the intraday period *d* on trading day *t* and *CP*_*t*,*d*_ is the closing price for the intraday period *d* on trading day *t*. Following [5] and [39], we calculate the bid-ask spread as the difference in prices;

$$BAS_{i}=\frac{ASK_{i}-BID_{i}}{(ASK_{i}+BID_{i})/2}$$ (2)

where *ASK*_*i*_ is the ask price of precious metal *i* and *BID*_*i*_ is the bid price of precious metal *i*. Given that true volatility is unobservable, the empirical results may be sensitive to the chosen volatility measure. In this paper, the intraday volatility is calculated using three approaches;

VOtSQ=  ln(CPtCPt−1)2 (3)

$$VO_{t}^{GK}=\;0.5\;{\left[ln\left(HP_{t}\right)-ln\left(LP_{t}\right)\right]}^{2}-\left[2ln2-1\right]{\left[ln\left(CP_{t}\right)-ln\left(OP_{t}\right)\right]}^{2}$$ (4)

$$VO_{t}^{RS}=[ln\left(HP_{t}-ln\left(OP_{t}\right)\right]\left[ln\left(HP_{t}\right)-ln\left(CP_{t}\right)\right]+[ln\left(LP_{t}\right)-ln\left(OP_{t}\right)]\left[ln\left(LP_{t}\right)-ln\left(CP_{t}\right)\right]$$ (5)

Where $VO_{t}^{SQ}$, $VO_{t}^{GK}$ and $VO_{t}^{RS}$ are the square return, volatility proposed by [40], and the volatility of [41] and [42]. *HP*, *LP*, *CP* and *OP* represent the high price, low price, closing price and opening price respectively. These different measures of volatility are calculated in different ways and therefore may provide differing results. The GK and RS measures that take into account the high, low, opening and closing prices of the precious metals when calculating volatility while the SQ measure just takes into account the returns of the precious metals. The GK and RS measures guard against the potential distorting impact of high-frequency real-world frictions by incorporating range information in the estimation of volatility, while the SQ measure does not. Therefore, although all three measures do calculate volatility, they do so in a slightly different manner and consequently may provide contrasting results.

**Fig 1:**
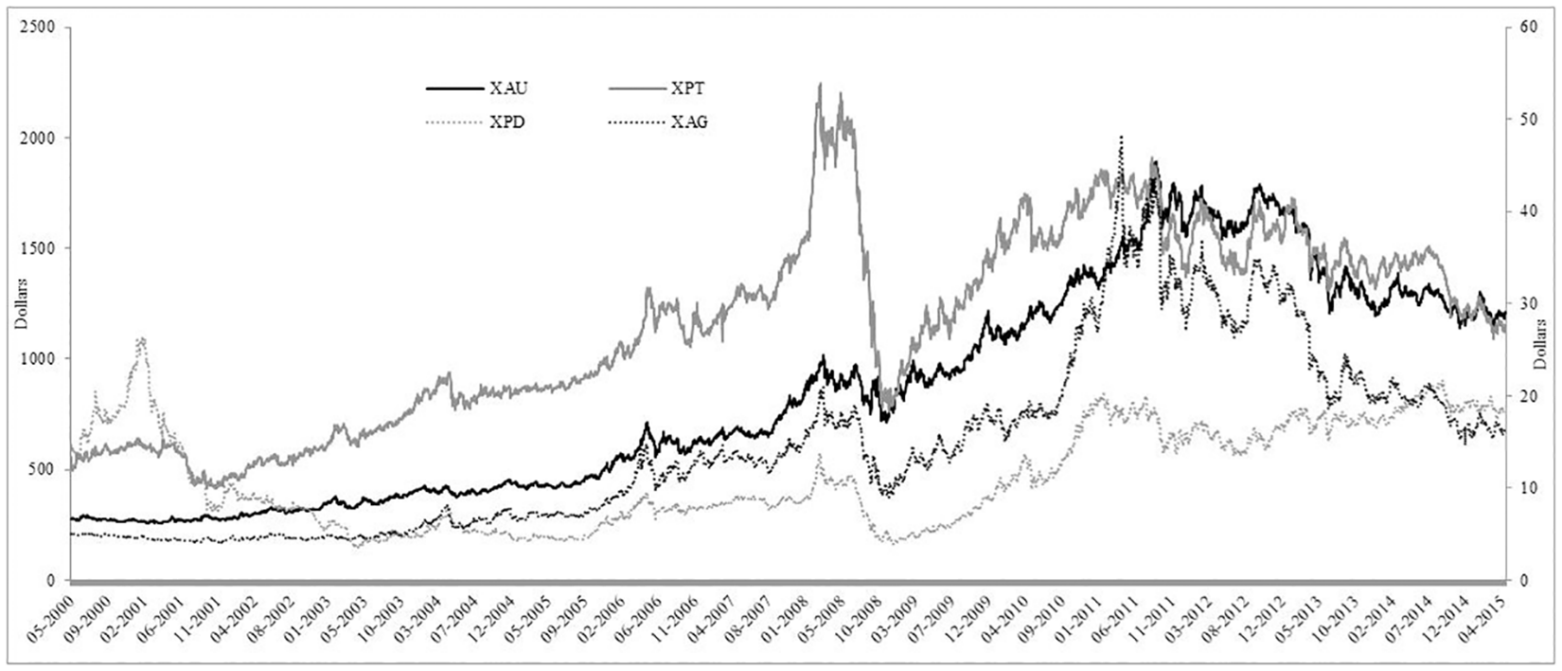
Time-series graphs of the prices of the four precious metals where XAU, XPT and XPD are on the primary y-axis and XAG is on the secondary y-axis.

**Fig 2:**
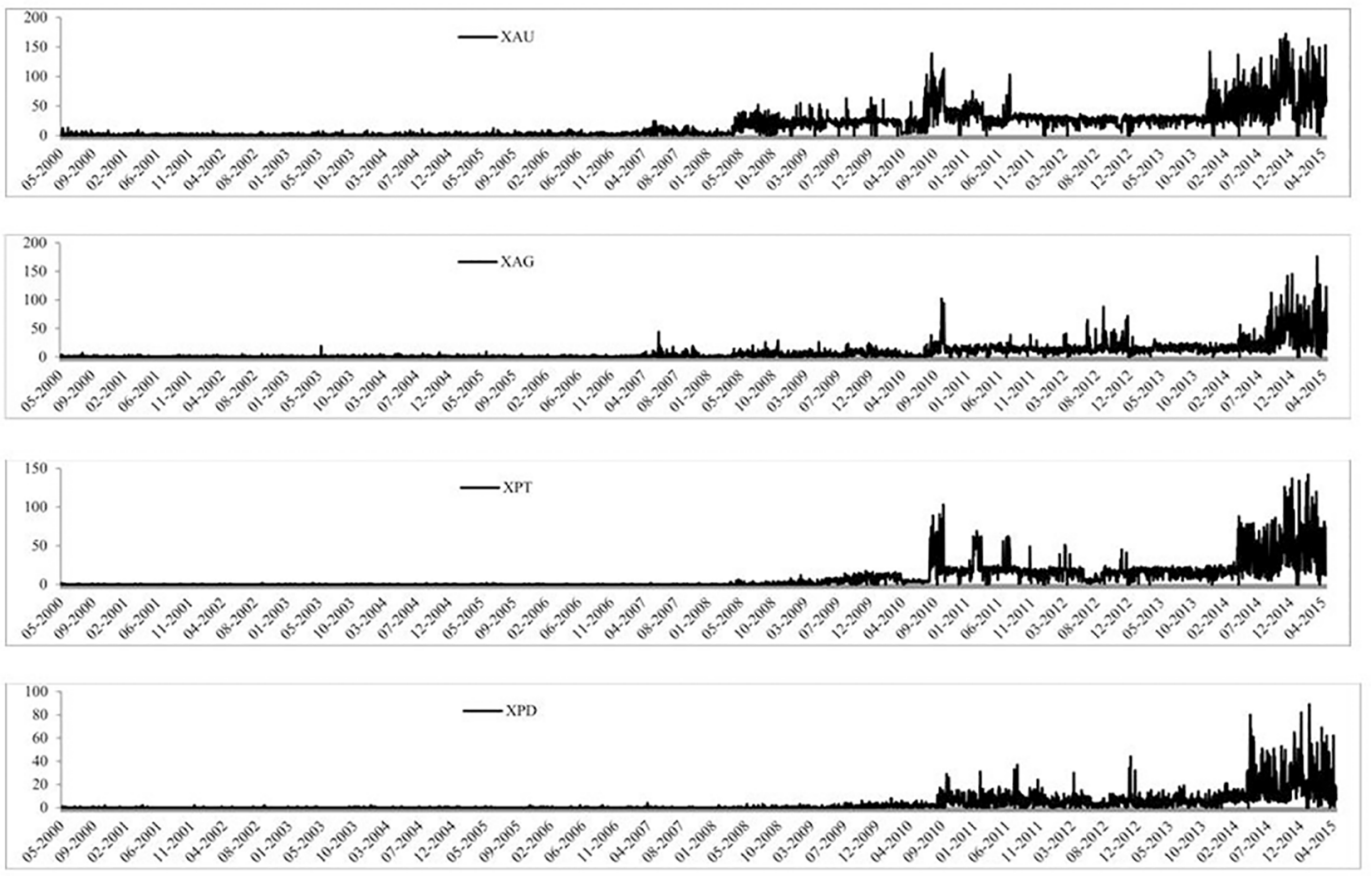
Time-series graphs of the volume of trades of the four precious metals.

**Fig 3:**
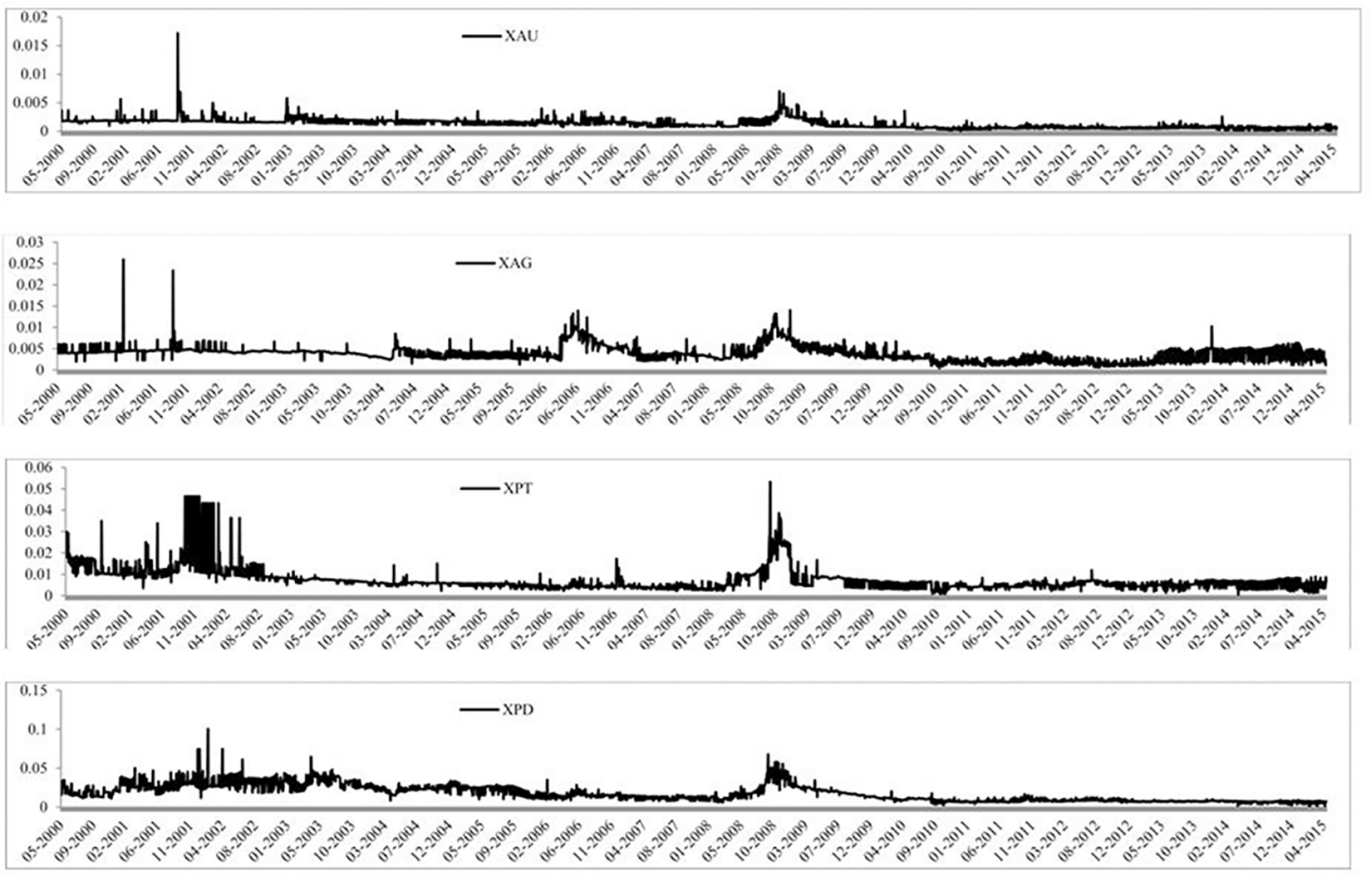
Time-series graphs of the BAS of the four precious metals.

**Fig 4:**
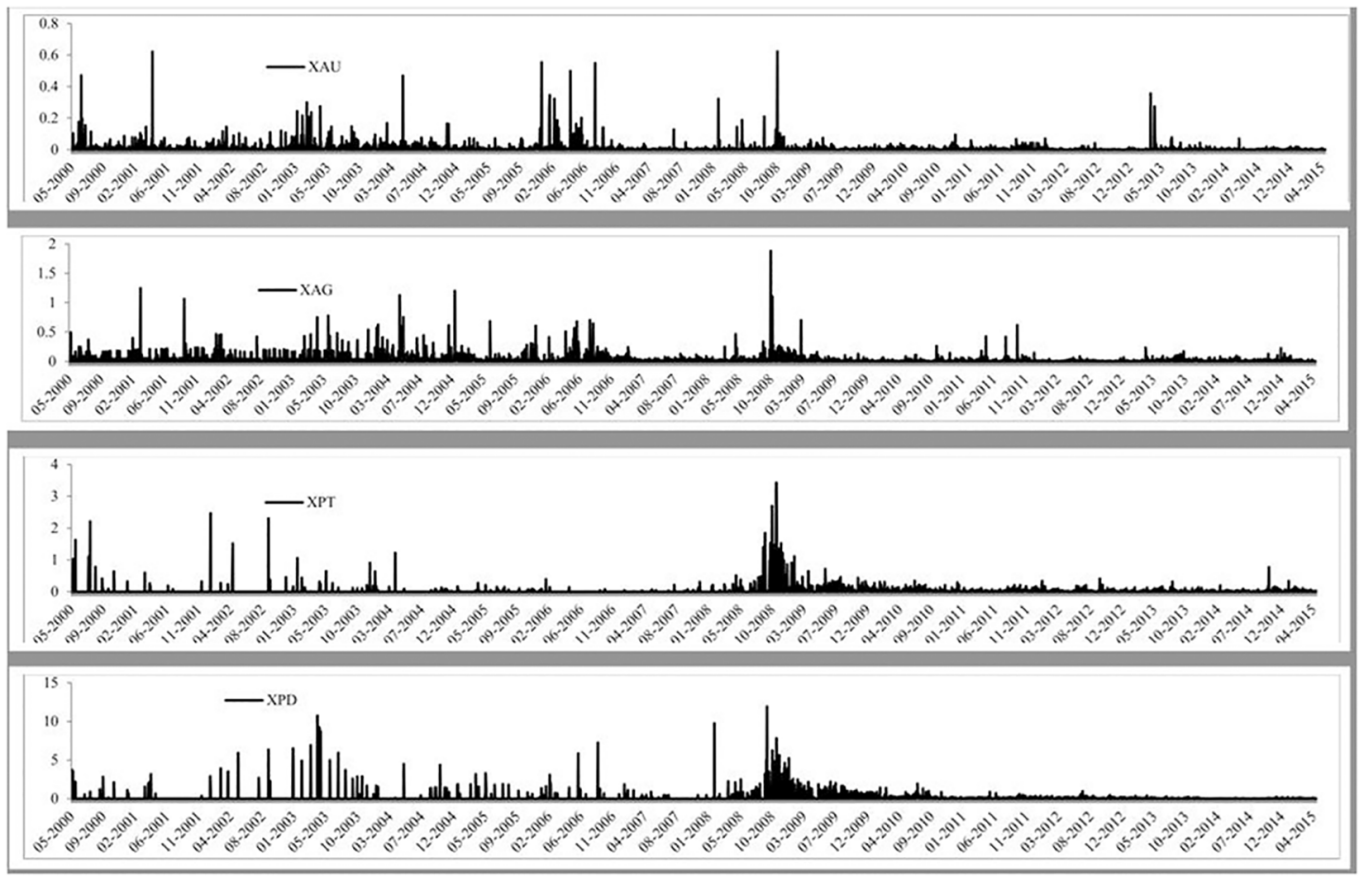
Time-series graphs of the squared returns measure of volatility of the four precious metals.

The time-series graph of each of the precious metals prices is reported in Fig 1, where the four precious metals seem to follow a similar pattern over time. We can see that silver has been very volatile and that palladium’s value is much less than the other three precious metals. Gold and silver have followed very similar paths since 2012 and that all four were affected by the 2008 global financial crisis. Fig 2 presents the volume of trades of each precious metal over time and we can see that each precious metal experiences a large increase in the number of trades throughout the sample period. It is also evident that gold has the largest volume of trades, followed by silver, platinum and palladium, which is also reported in Tables 1 and 2. The BAS are reported in Fig 3 and all precious show a large BAS at the beginning of the sample period. The spread does decreases after May 2003 for all precious metals and stays low throughout the sample period, except a sharp increase in the spread during the 2008 global financial crisis. Fig 4 reports the squared returns measure for volatility and shows that volatility for each precious metal was highest during the 2008 global financial crisis and at certain points in the early 2000s. Volatility is relatively low from 2010 to 2015, which may be due to the increase in volume of trading and thus efficiency.

**Table 1:** Descriptive statistics for the full sample gold, silver, platinum and palladium. ‘SQ’ denotes the squared returns measure of volatility, ‘GK’ denotes the Garman-Klass measure while ‘RS’ denotes the Rogers-Satchell measure.

	XAU	XAG	XPT	XPD
Panel A: Returns				
Mean	0.0000901	-0.0000231	-0.0000507	-0.0001706
Std	0.0796037	0.162851	0.2070907	0.4134391
Kurt	85.36	67.57	212.65	44.37
Skew	-0.6	-1.14	-1.25	-0.99
5% quant	0.0306469	-0.244998	-0.271639	-0.4814728
25% quant	0.1053416	-0.0516929	0	0
50% quant	0	0	0	0
75% quant	0.0306469	0.0579207	0.0059419	0
95% quant	0.1053416	0.229095	0.2757941	0.558661
Panel B: Vol^SQ^				
Mean	0.0000006	0.0265204	0.0428865	0.1709317
Std	0.0000059	0.2211995	0.6283358	1.1639475
Kurt	82650.17	89129.77	79533.57	129184.22
Skew	227.69	240.32	249.7	258.3
5% quant	0	0	0	0
25% quant	0	0	0	0
50% quant	0.0000001	0.0030575	0	0
75% quant	0.0000005	0.0227153	0.0143255	0.017405
95% quant	0.0000023	0.1054145	0.1758024	0.9005991
Panel C: Vol^GK^				
Mean	0.0075703	0.0152828	0.0127282	0.0142494
Std	0.0265717	0.037425	0.0164499	0.0213277
Kurt	88192.25	36409.7	115.87	42.3
Skew	283.82	173.29	4.74	3.81
5% quant	0	0	0	0
25% quant	0.0026343	0.0028133	0	0
50% quant	0.0063165	0.0124145	0.0063408	0.0040314
75% quant	0.010535	0.0233759	0.0223845	0.0242976
95% quant	0.0192804	0.0409709	0.0415909	0.051246
Panel D: Vol^RS^				
Mean	0.007523	0.0150185	0.0125224	0.0125509
Std	0.0370348	0.0159083	0.0169529	0.0212931
Kurt	93433.62	74.61	17.39	52.94
Skew	296.35	2.84	2.1	3.52
5% quant	0	0	0	0
25% quant	0.001585	0	0	0
50% quant	0.0063565	0.0125744	0	0
75% quant	0.0107109	0.0245575	0.0237169	0.0210129
95% quant	0.0197609	0.042643	0.0442581	0.0533983
Panel E: Volume				
Mean	21.9704	13.2289	6.1089	3.7997
Std	27.2509	22.2185	11.6664	106.15
Kurt	3.36	2082.75	4842.18	121075.1
Skew	1.64	12.47	20.91	346.2
5% quant	0	0	0	0
25% quant	1	0	0	0
50% quant	10	3	0	0
75% quant	37	19	9	4
95% quant	75	52	27	17
Panel F: BAS				
Mean	0.0012838	0.0038627	0.0068356	0.0173066
Std	0.000765	0.0176961	0.010543	0.0109459
Kurt	162.94	12592.23	30361.7	3988.74
Skew	4.97	111.66	-146.85	22.96
5% quant	0.000438	0.0012642	0.0029789	0.0066687
25% quant	0.0006769	0.0025233	0.004324	0.0084034
50% quant	0.0012001	0.0037922	0.0057904	0.0148368
75% quant	0.0017833	0.0044623	0.0081533	0.0234192
95% quant	0.0023684	0.0069136	0.0149254	0.0377358
Obs	1,079,830	1,079,750	1,079,679	1,079,688

**Table 2:** Descriptive statistics for gold and silver over the three subsamples. ‘SQ’ denotes the squared returns measure of volatility, ‘GK’ denotes the Garman-Klass measure while ‘RS’ denotes the Rogers-Satchell measure.

	XAU			XAG		
	2000–2005	2005–2010	2010–2015	2000–2005	2005–2010	2010–2015
Panel A: Returns						
Mean	0.0001539	0.0001586	-0.0000423	0.0001313	-0.000046	-0.0001547
Std	0.0781752	0.0911624	0.0677261	0.1545071	0.1856633	0.1456374
Kurt	43.64	106.39	60.87	81.44	36.9	115.62
Skew	-0.52	-0.94	0.16	-0.98	-1.2	-1.14
5% quant	-0.131098	-0.1290822	-0.0926088	-0.233918	-0.291971	-0.207361
25% quant	-0.0139772	-0.0329164	-0.0260909	0	-0.0743218	-0.0621311
50% quant	0	0	0	0	0	0
75% quant	0.0280181	0.0369622	0.0264651	0	0.0783392	0.0619195
95% quant	0.100007	0.1232224	0.0927663	0.223464	0.277393	0.208877
Panel B: Vol^SQ^						
Mean	0.0061114	0.0083106	0.0045868	0.0238724	0.0344708	0.0212102
Std	0.0412832	0.0865177	0.0363694	0.2180535	0.2150014	0.2300389
Kurt	22665.23	52354.51	27800.73	48209.64	46520.06	154210.01
Skew	119.76	196.31	129.54	185.24	174.42	340.88
5% quant	0	0	0.0000004	0	0	0
25% quant	0	0.0001551	0.0001293	0	0	0.0009529
50% quant	0.0005131	0.0012179	0.0006903	0	0.0058316	0.0038483
75% quant	0.0062739	0.005627	0.0027779	0.0255591	0.0288249	0.0164745
95% quant	0.0230615	0.0308749	0.0166092	0.0955546	0.1418637	0.0761584
Panel C: Vol^GK^						
Mean	0.0042371	0.0089173	0.0095536	0.0072037	0.0147717	0.0238685
Std	0.0056034	0.0444597	0.0095908	0.0491881	0.014154	0.038006
Kurt	11.53	33674.9	16676.48	26452.65	9.36	28674.04
Skew	2.39	180.48	106.98	155.4	2.18	160.03
5% quant	0	0	0.0034378	0	0	0.0066652
025% quant	0	0.0035623	0.0059895	0	0.0048847	0.0151101
50% quant	0.002106	0.006749	0.0085167	0	0.0114479	0.0224272
75% quant	0.0063465	0.0116234	0.0117261	0.0106443	0.0206488	0.0303305
95% quant	0.0153137	0.0228446	0.0190101	0.0294107	0.0406244	0.0450477
Panel D: Vol^RS^						
Mean	0.0038064	0.0089922	0.0097673	0.0056961	0.0146151	0.0247391
Std	0.0061344	0.062423	0.0125277	0.0134366	0.0152348	0.012847
Kurt	17.98	34649.13	22924.51	444.32	11.65	4.26
Skew	2.72	184.37	135.53	10.25	2.17	1.06
5% quant	0	0	0.0034769	0	0	0.0068632
25% quant	0	0.0033214	0.0061366	0	0	0.0159229
50% quant	0	0.0067261	0.008711	0	0.0116377	0.0236178
75% quant	0.0062108	0.0117933	0.0119516	0.0320555	0.0213779	0.0317143
95% quant	0.0159441	0.0233382	0.0192981	0.2578042	0.0420991	0.0467666
Panel E: Volume						
Mean	2.28512	16.5572	47.06131	0.9122	7.3949	31.3766
Std	4.93763	118.59424	28.48518	2.3133	10.7082	29.0856
Kurt	32.67	1.13	2.52713	148.16	6.5689	2088.77
Skew	4.51	1.31	1.23702	7.19	2.3733	14.62
5% quant	0	0	11	0	0	3
25% quant	0	2	27	0	0	14
50% quant	0	9	44	0	3	25
75% quant	2	26	59	1	10	39
95% quant	12	57	104	5	31	89
Panel F: BAS						
Mean	0.0018539	0.0013977	0.0006002	0.0043048	0.0044053	0.0028778
Std	0.0006416	0.0006908	0.0002486	0.0011092	0.0019853	0.0305432
Kurt	263.96	504.39	15.14	733.25	6.01	4263.46
Skew	11.68	8.94	2.33	15.35	1.72	65.25
5% quant	0.0012523	0.0007244	0.0002016	0.0028531	0.0023895	0.0010045
25% quant	0.0016095	0.0009029	0.0004774	0.0040241	0.0030143	0.0014489
50% quant	0.0018152	0.0012031	0.0005766	0.0043073	0.0037125	0.002007
75% quant	0.0018972	0.001642	0.0006846	0.0045351	0.005301	0.0028531
95% quant	0.0025924	0.0025238	0.0009878	0.0060423	0.0085561	0.0050865
Obs	359,772	360,180	359,928	359,676	360,180	359,894

## Results

This section provides the results for the stylized facts of gold, silver, platinum and palladium returns, volatility, volume and BAS.

### Full sample descriptive statistics

The descriptive statistics for the return series, the volatility measures, volume and BAS for the full sample period of the four precious metals are reported in Table 1. Panel A shows gold is the only precious metal to report a positive mean return over are sample period while platinum has the highest negative mean return and palladium the least negative mean return. Gold returns are also the least volatile of the precious metals while palladium is found to be the most volatile. This is consistent with the finding of [22] that gold has a larger interest than other precious metals that may lead to higher efficiency compared to other precious metals, which leads to smaller risk. The kurtosis of gold is much higher than other precious metals with silver having the lowest kurtosis. All precious metals have negative skewness, which is behaviour similar to that observed in equities.

Panels B, C and D of Table 1 report the descriptive statistics for the volatility measures of the four precious metals. The SQ measure suggests that platinum has the highest mean volatility, while the GK and RS measures both suggest that silver has the highest mean volatility. Gold has the highest positive kurtosis according to the GK and RS measures, while the SQ measure suggests that palladium has the highest kurtosis. All four precious metals volatility measures have positive skewness, with the SQ measure attributing the highest skewness to palladium, while the GK and RS measures suggest that gold has the highest skewness. Panel E of Table 1 reports the descriptive statistics of the volume of trades and shows that gold has the largest mean volume and palladium has the highest variation in volume, followed by silver, platinum and palladium. All four precious metals volume measures have excess kurtosis and positive skewness, which increases as the number of trades fall. The BAS analysis of the precious metals is reported in Panel F of Table 1 and shows that platinum has the largest mean spread, followed by silver, palladium and finally gold. Gold has the smallest mean standard deviation of BAS while silver has the greatest. The kurtosis of each precious metals BAS indicates leptokurtic distributions and positive skewness.

Overall, from the full sample analysis we can see that gold has the highest mean return and seems the most liquid since it has the highest mean volume and lowest mean BAS over the full sample. Palladium seems the least liquid precious metal with the lowest mean volume and highest mean BAS.

### Subsample descriptive statistics

In order to see how the stylized facts of these precious metals have behaved over our sample period, we split the full sample period into three equal sub-periods and repeat the analysis reported in Table 1. The results are reported in Table 2 for gold and silver and Table 3 for platinum and palladium.

**Table 3:** Descriptive statistics for platinum and palladium over the three subsamples. ‘SQ’ denotes the squared returns measure of volatility, ‘GK’ denotes the Garman-Klass measure while ‘RS’ denotes the Rogers-Satchell measure.

	XPT			XPD		
	2000–2005	2005–2010	2010–2015	2000–2005	2005–2010	2010–2015
Panel A: Returns						
Mean	0.0000261	-0.0002689	-0.0004081	-0.0003236	0.0001407	-0.0003292
Std	0.2316635	0.1313224	0.1325972	0.4563751	0.5106873	0.2089926
Kurt	65.16	12.4	10.22	21.24	42.34	4.9
Skew	-0.31	-0.27	-0.22	-1.3	-0.64	-0.1664
5% quant	-0.3243761	-0.2131644	-0.2161918	0	-0.835078	-0.3611549
25% quant	0	-0.0642675	-0.0650618	0	0	-0.0831324
50% quant	0	0	0	0	0	0
75% quant	0	0.065083	0.0644745	0	0	0.082306
95% quant	0.288123	0.206541	0.215728	0.508907	0.805806	0.369086
Panel B: Vol^SQ^						
Mean	0.0536678	0.0172456	0.0175821	0.2082778	0.2608008	0.0436779
Std	0.4398046	0.0654449	0.0614795	1.0040702	1.7365975	0.1147294
Kurt	82256.58	5901.34	7095.85	516.42	78042.21	2210.22
Skew	216.23	59.3982271	59.76	14.96	230.06	25.4568
5% quant	0	0	0	0	0	0
25% quant	0	0.0006522	0.000581	0	0	0.0009081
50% quant	0	0.0041785	0.0041947	0	0	0.0068422
75% quant	0.0133805	0.0188734	0.018502	0	0.0474652	0.0411502
95% quant	0.2083128	0.0700132	0.0743775	1.4692834	1.2913595	0.1969263
Panel C: Vol^GK^						
Mean	0.0064574	0.0230374	0.0252609	0.0052386	0.0115267	0.0259776
Std	0.0121131	0.0107706	0.0125457	0.0184928	0.0230601	0.0162669
Kurt	9.75	9.16	4.06	141.37	43.15	-0.0415584
Skew	2.72	1.45	1.0734	8.48	4.76	0.5461874
5% quant	0	0.0073931	0.0076231	0	0	0.0034676
25% quant	0	0.0165241	0.0171216	0	0	0.0124797
50% quant	0	0.0221416	0.0234502	0	0	0.0244
75% quant	0.0072826	0.0284787	0.0317613	0	0.0149993	0.0374223
95% quant	0.0336807	0.041013	0.0480681	0.0312871	0.0538244	0.0541242
Panel D: Vol^RS^						
Mean	0.0054296	0.0247651	0.0273644	0.0015561	0.0084784	0.0276124
Std	0.0125796	0.0125911	0.014309	0.014508	0.0210822	0.0184613
Kurt	12.79	16.75	8.43	711.29	20.74	0.1865454
Skew	3.1	2.08	1.49	19.1	3.72	0.5839
5% quant	0	0.00599645	0.0070885	0	0	0
25% quant	0	0.0176513	0.0184945	0	0	0.0125471
50% quant	0	0.02397	0.0254553	0	0	0.0257088
75% quant	0	0.0307562	0.0343755	0	0	0.0406122
95% quant	0.0345248	0.0442156	0.0524275	0	0.0531628	0.0592283
Panel E: Volume						
Mean	1.1077	12.5458	15.9085	0.0752	0.6777	10.6456
Std	3.4527	10.8855	15.1886	0.3649	1.9821	183.6478
Kurt	242.87	10.64	4983.8	499.9	55.3419	40505.6
Skew	9.87	2.45	25.95	12.31	5.8662	200.43
5% quant	0	0	1	0	0	0
25% quant	0	5	7	0	0	3
50% quant	0	11	13	0	0	7
75% quant	1	16	19	0	0	13
95% quant	7	32	47	1	4	32
Panel F: BAS						
Mean	0.006409	0.0049555	0.0050733	0.0267059	0.0172727	0.0079489
Std	0.0049016	0.0049242	0.0049665	0.0084668	0.0079979	0.0069243
Kurt	17.04	151108.09	146149.84	2.8085356	6.83	76128.69
Skew	3.47	-2.76	-2.76	1.1	2.18	264.65
5% quant	0.0030817	0.0028241	0.0025684	0.0149925	0.00907803	0.0048251
25% quant	0.004008	0.0040628	0.0041728	0.0210526	0.01204822	0.0070274
50% quant	0.004761	0.0048997	0.0050234	0.0254453	0.0152091	0.0076211
75% quant	0.0059701	0.0061425	0.0063336	0.0309598	0.01988078	0.0085616
95% quant	0.0168138	0.0071136	0.0073651	0.0424328	0.0322581	0.0114811
Obs	359,727	360,180	359,902	359,606	360,180	359,902

Table 2 reports the sub-sample analysis of the descriptive statistics of gold and shows that the 2005–2010 period had the largest mean return, while the 2010–2015 period had a negative mean return. The 2005–2010 period also had the largest standard deviation, the highest kurtosis and largest negative skewness of the three sub-samples. In the 2010–2015 period for gold, returns were positively skewed compared to negative skewness in the previous two periods, indicating that the gold returns in the 2010–2015 period behaved differently to the previous periods. The SQ and GK volatility measures show that volatility increased from the first subsample to the second subsample, but in the final subsample the volatility is at its lowest. The RS measure, however, suggests that volatility has increased over time in each subsample period. The volume results show that the number of trades for gold has increased substantially over time, from 2.28 in the first subsample to 47.06 in the third subsample indicating the increase in trading in gold over the previous 15 years. The mean BAS has also decreased substantially over time, from 0.00185 in the 2000–2005 period to 0.000600 in the 2010–2015 period, also indicating an increase in liquidity and efficiency of the gold market. This finding is consistent with a number of other empirical studies.

The silver sub-sample results show that in the first period silver had a positive mean return, which turned negative in the middle period and increasingly negative in the final period. The 2005–2010 period has the largest standard deviation of returns, while the 2010–2015 period experiences the largest kurtosis of returns. All periods experience negative skewness with the 2005–2010 period experiencing the largest negative skewness. The SQ volatility measure suggests that the 2005–2010 subsample has the highest mean volatility, while the GK and RS measures both suggest that volatility has increased over time with the final subsample exhibiting the largest volatility. Similar to gold, the mean volume of trades of silver increases over time, from 0.91 in the 2000–2005 period to 31.38 in the 2010–2015 period indicating an increase in liquidity over time. Furthermore, the BAS has decreased over time from 0.00430 in the 2000–2005 period to 0.00288 in the 2010–2015 period, again suggesting an increase in liquidity and efficiency in the silver market.

The sub-sample platinum results are reported in Table 3, the largest mean return is in the 2000–2005 period while the other two subsamples have negative mean returns. The first period has the largest standard deviation of returns and all the returns have positive kurtosis and negative skewness, with the 2000–2005 period having the largest negative skewness. All three volatility measures suggest that the 2000–2005 period has the largest mean volatility and the 2005–2010 subsample is the least volatile period. The mean volume of platinum increases over time from 1.11 in the 2000–2005 period to 15.91 in the 2010–2015 period indicating a substantial increase in liquidity over time. Also, the BAS decreased from 0.0064 in the 2000–2005 subsample to 0.0050 in the 2005–2010 subsample. The BAS in the final subsample is slightly higher at 0.0051, indicating that from the first subsample to the final two the BAS has decreased, consistent with an increase in liquidity and efficiency of the platinum market.

The palladium results show that the 2000–2005 and 2010–2015 periods have negative mean returns, while the 2005–2010 period has a positive mean return. The 2005–2010 period experiences the largest standard deviation of returns and all periods have positive kurtosis and negative skewness. The SQ and RS measures of volatility indicate that the 2005–2010 period has the highest mean volatility while the GK measure suggests the 2010–2015 period has the highest volatility. The mean volume of trades increases substantially over time, from 0.08 in the 2000–2005 subsample to 10.65 in the 2010–2015 subsample. The BAS has decreased over time, from 0.02670 in the 2000–2005 period to 0.00795 in the 2010–2015 period, indicating an increase in liquidity and efficiency of the palladium market.

The sub-period results show that each precious metal experienced negative mean returns in the 2010–2015 period and that the number of trades increased substantially over time. Furthermore, the trading volume in the first sub-sample period is very low for each precious metal, indicating the lack of liquidity at the 5-minute level. Therefore our results show that the behaviour of precious metals has changed substantially over time.

### Intraday stylized facts

**Fig 5:**
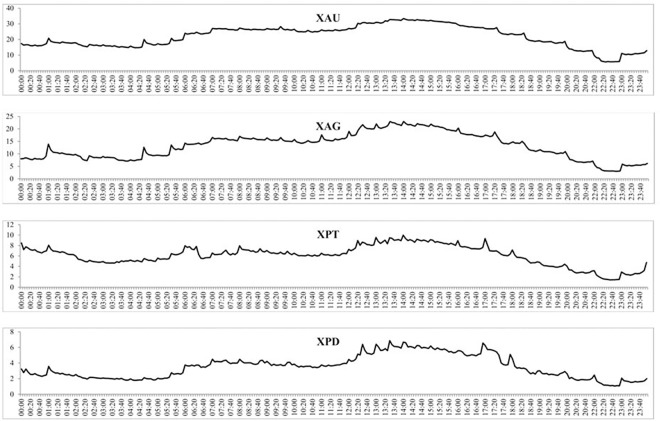
The mean volume of trades for each 5-minute period over the full sample of each precious metal.

Fig 5 reports the intraday mean volume of trades at the 5-minute intervals, all four precious metals exhibit n-shaped patterns, the number of trades increases until the early afternoon GMT and then falls away. This is consistent with the opening hours of European markets (9 AM to 5 PM GMT) and North American markets (about 3 PM to 8 PM GMT), where the highest volume of trades takes place round 11 AM GMT to 5 PM GMT when both markets are open. These findings suggest the possible presence of a periodic pattern in volume, which is investigated in more detail on the subsample level.

**Fig 6:**
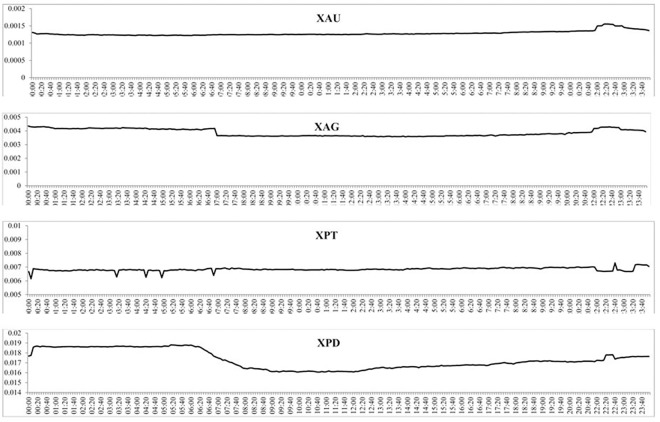
The mean BAS for each 5-minute period over the full sample of each precious metal.

**Fig 7:**
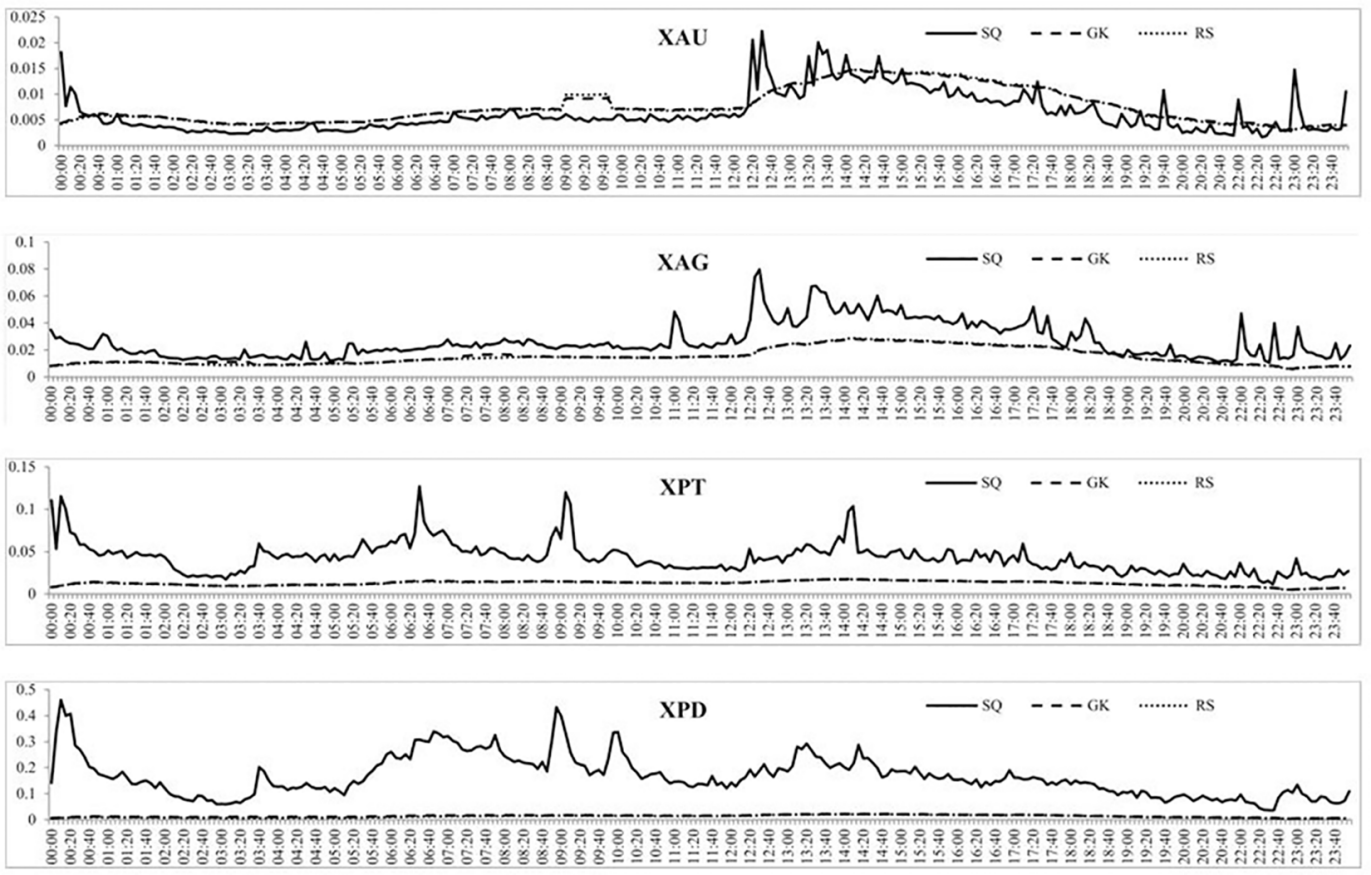
The mean volatility for each 5-minute period over the full sample of each precious metal employing the three different measures of volatility.

The intraday mean BAS at the 5-minute intervals for each precious metal are reported in Fig 6 and show that the mean BAS for gold and silver is fairly constant throughout the day. Both markets exhibit a small increase in the BAS around 10 PM GMT, possibly due to the daily hour closure of the markets from 9 PM GMT to 10 PM GMT. Platinum also shows a fairly constant BAS throughout the day with some very small fluctuations around 10 PM GMT. Palladium however exhibits some periodicity, with the BAS largest from midnight GMT to 6 AM GMT, which then falls and stays fairly constant until the end of the day, which could be the results of the opening (and anticipation) of European markets. Fig 7 reports the intraday volatility through the three volatility measures previously discussed and shows that the volatility for gold is fairly constant up to 12 PM GMT and then increases slightly until 2 PM GMT. After this point, volatility decreases and levels off to the end of the day. Silver’s volatility is fairly constant throughout the day, with again a small increase around 12 PM GMT which continues until 2 PM GMT. The GK and RS volatility for platinum and palladium are very similar and fairly constant throughout the day, while the SQ measure of volatility is little more variable with a few sharp jumps at various points of the day although there is no clear periodicity.

### Dynamic intraday stylized facts

As we have seen in Tables 2 and 3, the behaviour of the four precious metals has changed substantially over time and so their intraday behaviour may also change, depending on the sub period examined. Therefore we also study the dynamic intraday stylized facts in three subsamples to examine whether the behaviour of the precious metals markets change depending on the time period examined.

**Fig 8:**
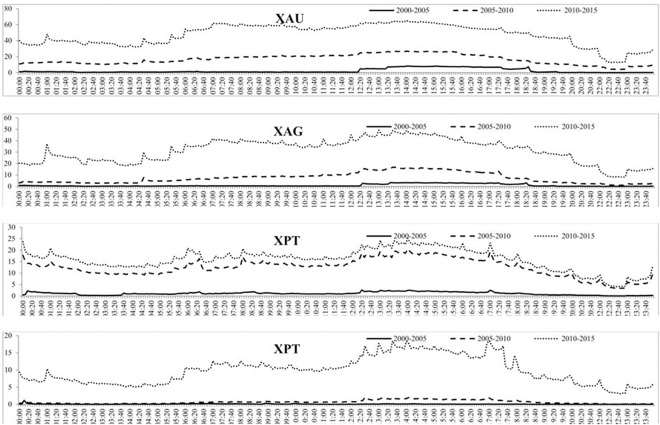
The mean volume of trades for each 5-minute period over the three subsamples for the four precious metals.

**Fig 9:**
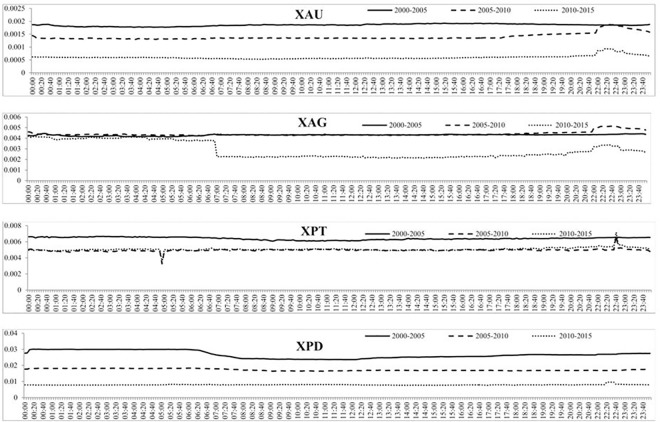
The mean BAS for each 5-minute period over the three subsamples for the four precious metals.

**Fig 10:**
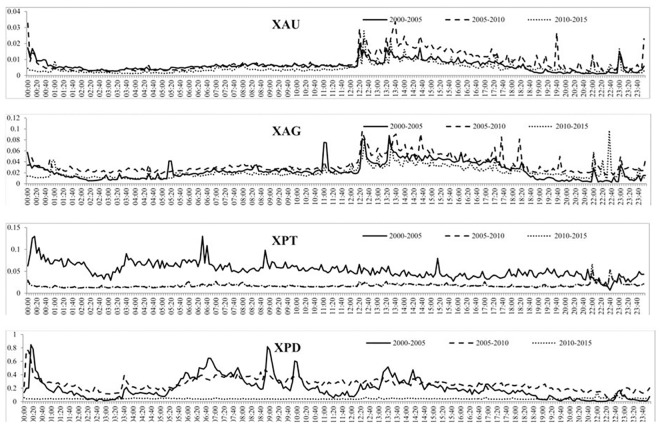
The mean squared returns measure of volatility for each 5-minute period over the three subsamples for the four precious metals.

Fig 8 shows the intraday volume of trades over the three subsamples and shows that each subsample experiences daily periodicity, albeit at different magnitudes. For instance, the volume of trades of gold increases throughout the day and then decreases from around 5 PM GMT, similar to what was found in Fig 5. For all four precious metals, the increase in the volume of trades is much larger from the second to the third subsample than the first to the second subsample, indicating a much larger increase in trading of precious metals after 2010. The intraday BAS over the three subsamples is reported in Fig 9 which, similar to the intraday BAS over the full sample, shows very little pattern throughout the day as the BAS seems to remain fairly constant in each subsample period. As expected from our previous analysis, the BAS of each precious metal decreases over time indicating an increase in liquidity and efficiency of each precious metal market. Fig 10 presents the intraday squared returns measure of volatility over the three subsamples and for gold and silver, the patterns are very similar. For platinum, we find that the volatility during the first subsample is much greater throughout the day than for the most recent subsamples while we find that the most recent subsample for palladium experiences much less variation throughout the day than the first two subsamples The other measures of volatility show similar patterns and are not reported to conserve space but available upon request.

### Correlation

A well-known stylised fact in finance is that stock index returns are negatively correlated with changes in volatility [26] and that the relationship is even more pronounced in falling than in rising markets [43]. There has been much evidence of this relationship in stock market indices but little in precious metals, especially at high-frequency. To examine the lead-lag relationship between returns and volatility of returns, we calculate the correlation coefficient of the precious metals returns at the 5-minute internal *t* with VSQ and VGK in 5-minutes internal *t + j*, where *j* ∈ {-500,…,500}. Calculations are based on all *t* during the total sample period. The RS graphs are almost identical to those of the GK and are not included but are available upon request.

**Fig 11:**
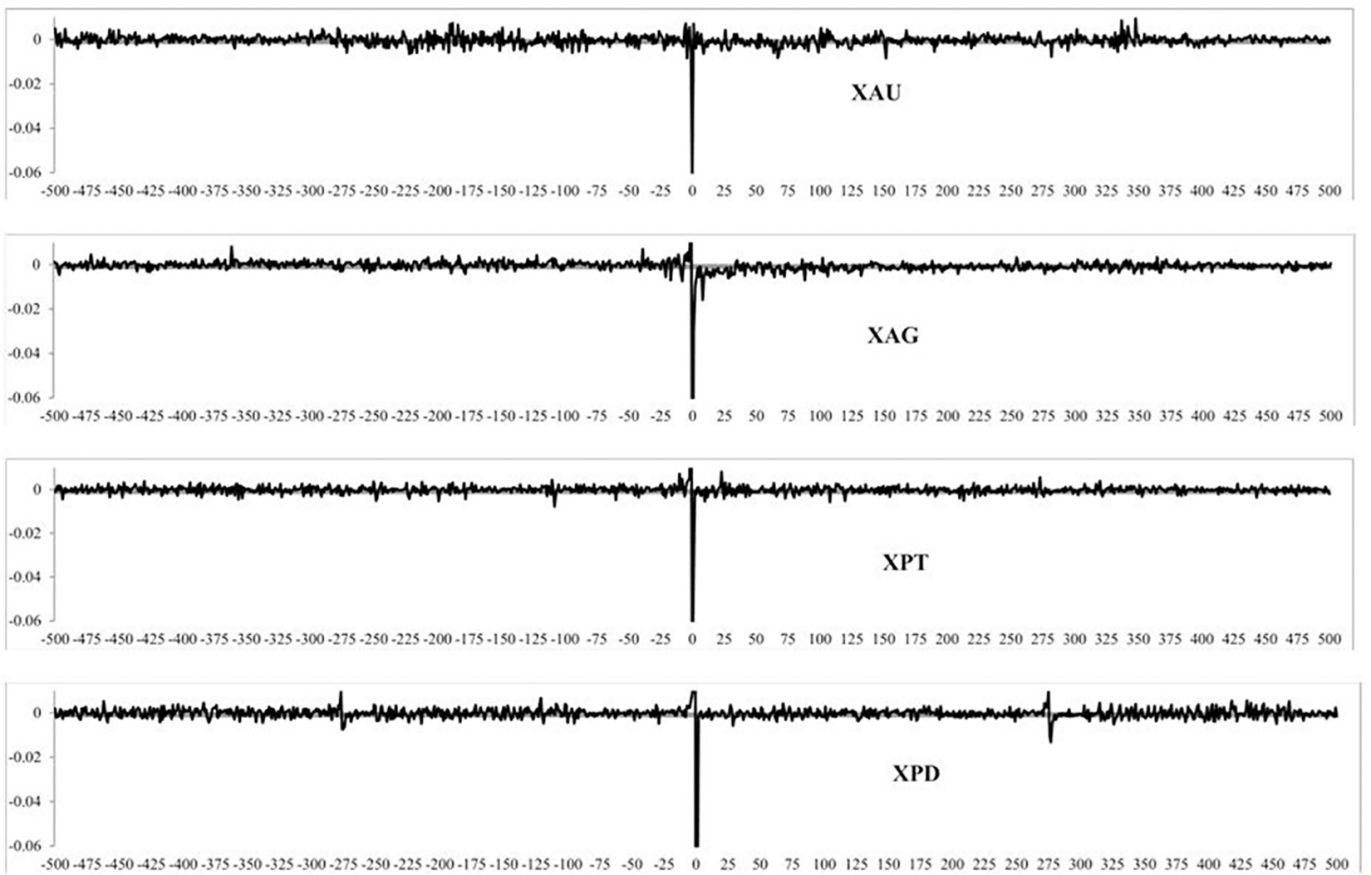
The correlation between returns and volatility, measured by squared returns over the full sample period for different lead and lag intervals.

**Fig 12:**
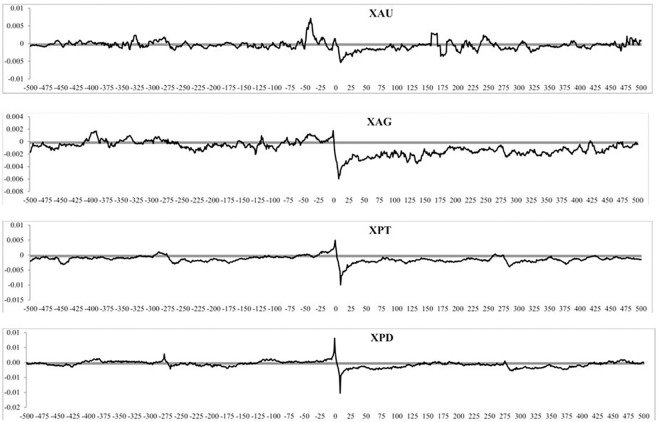
The correlation between returns and volatility, measured by the Garman and Klass (1980) measure over the full sample period for different lead and lag intervals.

Fig 11 shows the correlation coefficient is near zero for lagged SQ volatility (*j < 0*) for all precious metals. Thus, precious metals return does not seem to be systematically related to the preceding SQ volatility. However, we find a significantly negative correlation for all four precious metals returns not only with contemporaneous volatility (*j = 0*), but also the volatility in the next few 5-minute periods. This observation supports the hypothesis that volatility is adjusted to changes in the index level. We also study the GK volatility measure interaction with returns in Fig 12, which shows similar results to Fig 11, with lagged volatility generating near zero coefficients and some significant negative correlation coefficients. Again, this is compatible with a return-driven effect. We also study the correlation between volatility and returns for our three subsamples and find them to be almost identical to ones reported in Figs 11 and 12 and are available upon request. However, correlations computed at lags > 1 could be due to the correlation at lag *j = 1*. This is why it is necessary to identify causality and the number of lagged returns which have an impact on contemporaneous volatility.

### Vector autoregression model

To explore the casual relationships between volatility and returns of high-frequency precious metal data, a vector autoregression (VAR) model is estimated. Granger causality tests are then conducted to determine the direction of the causal linkages.

We consider a VAR model of order *p* in which;

$$y_{t}=c+\overset{p}{\underset{t=1}{\sum^{\text{​}}}}\phi_{i}y_{t-1}+\varepsilon_{t}$$ (6)

where *y*_*t*_ is a (*n × 1*) vector of endogenous variables, *c = (c*_*1*_,… *c*_*n*_) is the (*n × 1*) intercept vector of the VAR,*ϕ*_*i*_ is the *i*th (*n × n*) matrix of autoregressive coefficients for *i = 1*, *2*,…, *p*, and *ε*_*t*_ = (*ε*_1*t*_,…*ε*_*nt*_) is the (*n × 1*) generalization of a white noise process. We model the return volatility relationships across the four precious metals where the models are estimated up to a maximum lag of 12 and the optimal lag length is selected by using the Akaike information criterion (AIC), similar to [44].

After estimating the VAR model, the Granger causality test is conducted, this is a popular way to test if there is any temporal statistical relationship with a predictive value between the two time series [45]. This test indicates any possible short-run predictive interrelationships among the series. When ‘X Granger causes Y’, it does not mean that Y is the effect or the result of X. Granger causality measures precedence and information content and thus ‘causality’ is defined in terms of predictability, hence variable X causes variable Y if present Y can be better predicted by using past values of X than by not doing so, with respect to a given information set that includes X and Y.

Table 4 summaries the results of the Granger causality test for the full sample period as well as the three subsample periods for the return-driven relationship in Panel A and the volatility-driven relationship in Panel B. The results clearly show strong evidence of a return-driven relationship across all sample periods and all three measures of volatility for platinum and palladium. For gold we find significant evidence of a return-driven relationship at the 5% level for all measures and sample periods except the GK and RS measures in the 2005–2010 period, where the return-driven relationship is only significant at the 7% level. For silver, we find significant evidence of a return-driven relationship for all sample periods for all SQ and RS volatility measures but find insignificant evidence for the GK measure in the 2000–2015, 2000–2005 and 2010–2015 periods. We also find significant evidence of a volatility-driven relationship since all p-values are significant at the 5% level. That means that past volatility does add significant explanatory power of past returns in explaining current returns. This relationship is consistent across sample periods and across measures for volatility. Therefore we provide evidence of a bi-lateral relationship between returns and volatility of precious metals, in that returns and volatility both have strong explanatory power in explaining current volatility and current returns.

**Table 4:** The Granger causality test result p-values for the full sample and three subsamples of the return-volatility relationships. ‘SQ’ denotes the squared returns measure of volatility, ‘GK’ denotes the Garman-Klass measure and ‘RS’ denotes the Rogers-Satchell measure of volatility.

	2000–2015			2000–2005			2005–2010			2010–2015		
	SQ	GK	RS	SQ	GK	RS	SQ	GK	RS	SQ	GK	RS
Panel A: Return-driven relationship												
XAU	0	0.02	0.02	0	0	0	0	0.07	0.07	0	0.04	0.01
XAG	0	0.72	0	0	0.12	0	0	0.03	0.01	0	0.78	0
XPT	0	0	0	0	0	0	0	0	0	0.01	0	0
XPD	0	0	0	0	0	0	0	0	0	0	0	0
Panel B: Volatility-driven relationship												
XAU	0	0	0	0	0	0	0	0.04	0.05	0	0	0
XAG	0	0	0	0	0	0	0	0	0	0	0	0
XPT	0	0	0	0	0	0	0	0	0	0	0	0
XPD	0	0	0	0	0	0	0	0	0	0	0	0

## Discussion and conclusions

This study investigates the intraday periodicity, correlation and volatility interaction of returns, volatility, volume and BAS that occur in 5-minute data for the key precious metals: gold, silver, platinum and palladium. We study the intraday periodicity as well as the relationship between returns and volatility from 2000 to 2015, as well as in three subsamples to determine how the precious metals stylized facts have developed over time. These precious metals are some of the most traded assets worldwide and they also play an important role for investors as well as comprising an important asset for central banks. Given the increased attention precious metals have received in the literature, the intraday dynamics are of great interest.

Initially, we show that the volume of trades of precious metals has increased substantially over the last 15 years’ while the bid-ask spread has decreased indicating the increase in efficiency and liquidity of precious metal markets. We also show strong evidence of intraday periodicity of precious metals volume of trades and volatility. The intraday volume has increased over time, while the intraday bid-ask spread has decreased over time. The narrowing of bid-ask spreads and increased trading volume could partially be attributed to the global financial crisis of 2007–2009 and the subsequent European sovereign debt crisis since market participants may have chosen to gold as a safe haven or risk-hedging tool during this period (see [46] for more details). We also study the interaction between volatility and returns of each precious metal and our correlation analysis shows that returns are negatively correlated with the contemporaneous volatility and the previous 5-minute volatility. Furthermore, we find bi-directional Granger causality between volatility and returns suggesting that past volatility (returns) offers significant explanatory power in explaining current returns (volatility).
